# Seed Germination and Seed Bank Dynamics of *Eruca sativa* (Brassicaceae): A Weed on the Northeastern Edge of Tibetan Plateau

**DOI:** 10.3389/fpls.2022.820925

**Published:** 2022-03-10

**Authors:** Cun-Zhi Jia, Jing-Jing Wang, Da-Li Chen, Xiao-Wen Hu

**Affiliations:** ^1^State Key Laboratory of Grassland Agro-ecosystems, Lanzhou University, Lanzhou, China; ^2^Key Laboratory of Grassland Livestock Industry Innovation, Ministry of Agriculture and Rural Affairs, Lanzhou University, Lanzhou, China; ^3^Engineering Research Center of Grassland Industry, Ministry of Education, Lanzhou University, Lanzhou, China; ^4^College of Pastoral Agriculture Science and Technology, Lanzhou University, Lanzhou, China

**Keywords:** dormancy cycling, *Eruca sativa*, persistence of soil seed bank, weed management, Qinghai-Tibet Plateau

## Abstract

As a versatile cruciferous species, *Eruca sativa* is widely cultivated, but in some areas, it has become an invasive weed. There are few studies on its seed dormancy and soil seed bank. This research examined seed dormancy, germination, and dynamics of the soil seed bank of *E. sativa*, with a view to provide support for its prevention and control. We tested the effects of temperature, light, storage, water, and salinity stress on seed germination and burial depth on seedling emergence of *E. sativa*. Dynamics of the soil seed bank were determined with a 24 month *in situ* seed-burial study. Seeds of *E. sativa* can germinate in a temperature range of 5–35°C; moreover, they exhibited non-deep physiological dormancy (NDPD) at maturity, which can be broken by dry storage or exposure to low temperature in winter. Germination of *E. sativa* seeds was sensitive to water and salinity stress, and most seeds did not germinate at -0.3 MPa. When buried in soil in the field, seeds exhibited an annual dormancy/non-dormancy cycle and formed at least a short-term persistent soil seed bank. Seeds buried deeper than 5 cm can hardly emerge. Seeds of *E. sativa* have a wide germination temperature range and exhibited dormancy cycling, which promotes the formation of a persistent soil seed bank and enables it to better adapt to the harsh low-temperature climate of the Qinghai-Tibet Plateau. No-tillage would be a good management strategy for this species.

## Introduction

Seed dormancy, germination, and seedling establishment are crucial steps in the life cycle of seed plants (Bewley et al., 2013). A successful weed management program must take these aspects of weed biology and ecology into account (Forcella et al., 1993).

The successful establishment of a seedling depends largely on the interaction between germination requirements and the environment (Hilhorst, 2008). The ability to germinate successfully under a variety of environmental conditions is a characteristic of many successful and widespread weed species (Baker, 1991; Wainwright and Cleland, 2013). Temperature, moisture, and light are the most critical environmental factors, which affect not only the germination of seeds, but also the persistence of the soil seed bank (Baskin and Baskin, 1985b; Gutterman, 1994). Thus, understanding the germination ecology of weeds is essential for their management and limiting their spread to new sites.

Seed dormancy is the failure of viable seeds to germinate in a specified period of time under optimal conditions (Baskin and Baskin, 2004). Some seeds exhibit seed dormancy cycles to ensure that seedling emergence occurs during a favorable season, increasing their chances of survival in a seasonally varying environment (Baskin and Baskin, 1985a; Carter and Ungar, 2003; Cao et al., 2014). The majority of studies involving seed dormancy cycles have been conducted with species from temperate regions (Carter and Ungar, 2003; Handley and Davy, 2005; Cao et al., 2014), whereas less is known about seed dormancy cycles of species growing on the Qinghai-Tibet Plateau in China. The exception is the study by Wang et al. (2017) who first comprehensively demonstrated the regulation of germination timing by dormancy cycling on *Primula alpicola* and *Pedicularis fletcheri* on the Tibet Plateau.

Serving as pools of genetic material, soil seed banks enable a range of responses to environmental conditions and buffer populations against temporary adverse environmental conditions (Teo-Sherrell et al., 1996), thus becoming the primary source of new infestations of annual weeds in crop production systems (Buhler, 1999). Many weed communities are regulated by the soil seed bank (Buhler et al., 1997; Hu et al., 2017, 2018; Das and Das, 2018). The persistence of soil seed banks of different species varies greatly (Baskin and Baskin, 2014) and can be influenced by the depth of burial (Hu et al., 2014).

Rocket (*E. sativa* Mill.) is a summer annual herbaceous plant in the Brassicaceae, which is widely distributed in the temperate regions (Kanya and Urs, 1989). Meanwhile, due to its importance in industry, agriculture, and medicine, rocket has been widely cultivated (Alotaibi et al., 2020; Bell et al., 2020; Golkar and Bakhtiari, 2020; Khan et al., 2021; Piragine et al., 2021). However, in some places of China, rocket has become an invasive weed. In Inner Mongolia, Hebei, and Shanxi, rocket caused a 6–36% reduction in yield of *Sesamum indicum* (Fu et al., 2020). Furthermore, rocket also can be found in oat (*Avena sativa* L.) fields on the northeastern edge of the Tibetan Plateau. Due to its rapid growth ability and large seed yield, rocket caused a severe reduction in the yield of local oats and other crops.

There are few studies on *E. sativa* seed dormancy and germination and soil seed bank characteristics. As far as we know, only three reports focused on seed germination characteristics and soil seed bank of this species. One of them quantified the effect of environmental factors on seed germination of a cultivated *E. sativa* (Bakhshandeh et al., 2020), and the other two studied germination characteristics and relative contribution of the soil seed bank to maintain the aboveground genetic diversity of different populations (Barazani et al., 2012; Hanin et al., 2013). However, information on seed dormancy and soil seed bank dynamics of *E. sativa* is not available. To solve this problem, a long-term *in situ* research is necessary. Therefore, the seed germination and soil seed bank characteristics were studied through a 24-month *in-situ* research trial to answer the following questions: (i) What are the environmental requirements for dormancy break and germination? (ii) Do buried seeds undergo an annual dormancy cycle? (iii) Do buried seeds form a persistent soil seed bank? and (iv) From what soil depth can seedlings emerge? Based on the results of our research, we hope to provide support for the prevention and control of *E. sativa*.

## Materials and Methods

### Seed Collection

**FIGURE 1:**
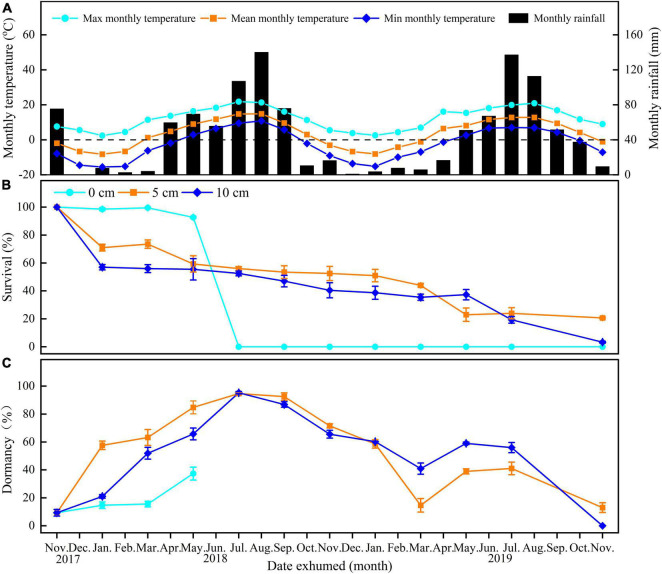
Mean maximum, mean minimum, and mean monthly temperature in Xiahe County, Gansu Province, China **(A)** and survival **(B)** and dormancy **(C)** of *E. sativa* seeds exhumed after burial for 0–24 months at different depths (on soil surface—0 cm, buried 5 cm in soil—5 cm, buried 10 cm in soil—10 cm). Error bars are ± 1 SE, *n* = 4. Temperature and rainfall data were obtained from the local government.

The study area is located in Xiahe County, Gannan Prefecture (35°14′N, 102°46 10 2,962 m a.s.l.), Gansu Province, China, which has a typical plateau continental climate. During the experiment, the annual precipitation was 500–550 mm, with over 80% of it falling during the growing season (i.e., April–September), and mean annual air temperatures were 3.62–3.79°C (Figure 1A). In this area, seeds of *E. sativa* mature in the beginning of August, and after seeds mature, some of them remain on the mother plants. We used two seed lots collected at different times: one was collected on August 20, 2017, in Ganga Town (hereafter, seed lot A), and the other one was collected on October 20, 2017, in Wanggertang Town (seed lot B). Both seed lots were collected from hundreds of individuals. Collected seeds were taken to the laboratory, cleaned and dried at room temperature [relative humidity (RH) 20–35%, 18–25°C] for 1 week, and then stored dry at 4°C until used in experiments that were conducted within 2 weeks after collection.

### Seed Mass

Thousand seed weight (TSW) was determined by weighing eight replicates of 1,000 seeds to the nearest 0.0001 g using a Sartorius electronic balance.

### Effect of Temperature and Light on Germination

To determine whether seeds are dormant at maturity, fresh seeds and seeds stored for 1 year of both seed lots were tested at 5, 10, 15, 20, 25, 30, 35, 40, 15/25, and 20/30°C (12 h/12 h) in light (12 h daily photoperiod) and in continuous darkness. For the two alternating temperature regimes, the higher temperature coincided with a 12 h light period and the lower temperature with a 12 h dark period. The light source was cold white fluorescent tubes, and photon irradiance was 60 μmol m^–2^s^–1^ (400–700 nm). All Petri dishes were wrapped in plastic film to reduce evaporation. For continuous darkness, Petri dishes were covered with two layers of aluminum foil. For each treatment, three replicates of 50 seeds were placed in Petri dishes of 11 cm diameter with two sheets of filter paper (Shuang quan, Hangzhou) moistened with 8 ml of distilled water. Germination of seeds incubated in light was monitored daily for at least 14 days until no further germination occurred for three consecutive days, and any seedlings present were counted and discarded. Seeds incubated in the dark were checked for germination only after 14 days. The emergence of the radicle (or cotyledons, which sometimes emerge from the seed coat before the radicle) was the criterion for germination in this and all other germination experiments. Following all germination tests, non-germinated seeds were examined under a dissecting microscope to determine whether the embryo was firm and white, indicating viability, or soft and gray, indicating non-viability. Only viable seeds were used in calculating germination percentages. The speed of germination, expressed as germination index (GI), was calculated using the following formula:


GI=∑(Gt/Tt)

where Gt is the number of seeds germinated on t-th day and Tt is the days of seed germination (Wang et al., 2004).

### Effect of Water Stress and Salinity on Germination

To determine the effect of water stress and salinity on seed germination, fresh seeds of both seed lots were incubated in six water potentials (i.e., 0, −0.3, −0.6, −0.9, and −1.2 MPa) with two osmotica (NaCl and polyethylene glycol [PEG], in reverse osmosis purified water) in light at 20°C. The osmotic potentials of these solutions were corrected for the effects of temperature using the relationships determined by Michel (1983). All Petri dishes were wrapped in plastic film to reduce evaporation, and the potential solution and filter paper were renewed every 2 days to keep the water potentials constant. Germination was monitored daily for 14 days. The GI was calculated as described earlier.

### Effects of Burial Depth on Seedling Emergence

The effects of the burial depth on seedling emergence were studied in a greenhouse located on the Yuzhong Campus with a daily temperature range of 18–22°C. Seeds of both seed lots were sown on the soil surface (0 cm) and at depths of 0.5, 1, 2, 3, and 4 cm. The soil used was from the seed collection site and was typical silt loam. The soil organic carbon, total nitrogen, and total phosphorus contents were 23.0, 2.0, and 1.5 g/kg, respectively, and the available nitrogen, phosphorus, and potassium contents were, respectively, 23.2, 23.1, and 305.0 mg/kg, and soil pH was 8.2. The same watering regime was used for all treatments, i.e., 80% field capacity. Four replicates with 100 seeds each were used for each burial depth. All plots were watered daily with tap water throughout the experimental period. Emerged seedlings (shoot visible at the soil surface) were counted and removed daily for 3 weeks. The speed of seedling emergence, which is expressed as seedling emergence index (EI), was calculated using the following formula:


EI=∑(Et/Tt)

where Et is the number of seedlings germinated on t-th day and Tt is the days of seedling emergence (Wang et al., 2004).

### Effect of Dry Storage on Germination

Fresh seeds of seed lot A were stored in a paper bag in darkness at 20°C (RH 20–35%) for 0, 2, 4, 6, 8, 10, and 12 months. After each storage period, the germination of four replicates of 50 seeds was tested in light at 20°C. This temperature and photoperiod conditions were used to simulate the indoor storage conditions of *E. sativa* seeds mixed in other crops after being harvested. Germination was monitored daily for 14 days as described earlier.

### Effect of Burial Depth on Persistence of Soil Seed Bank

Seed lot A was used to determine fates of seeds on the soil surface (0 cm) and those buried at 5 and 10 cm during a period of 24 months. The burial site was about 100 m from the seed collection site. During the test, weeds within the burial site were hand-removed weekly. On November 10, 2017, 144 nylon mesh bags (15 cm × 10 cm) containing 50 fresh seeds were buried, 48 bags were placed on the soil surface, and 48 at the depth of 5 and 10 cm each, in a randomized block design. Bags on the soil surface were fixed to the ground with iron nails so that each bag was in contact with the soil. After the burial, every 2 months, four bags from each depth (0, 5, and 10 cm) were exhumed for the germination experiment until November 2019. Seeds in each of the 12 bags were put into a Petri dish of 11 cm diameter with two layers of moist filter paper and incubated in light at 20°C. The mean optimal temperature for germination of arctic and alpine tundra species is 18.0 ± 0.3°C (Baskin and Baskin, 2014), thus, 20°C was chosen to simulate the germination environment. The number of germinated, dormant (non-germinated but viable), and dead (decayed or empty) seeds was counted after 14 days. Seeds that failed to germinate but had a firm white embryo were considered to be viable. The number of surviving seeds was obtained by adding the number of dormant seeds and the number of seeds that germinated. The percentage of dormancy was obtained by dividing the number of dormant seeds by the number of surviving seeds.

### Data Analysis

The effects of storage, light, incubation temperature, and their interaction on seed germination and the effects of burial depth, burial duration, and their interaction on percentage of survival and dormancy of seeds were tested by fitting generalized linear mixed models (GLMMs). Storage, temperature, light, burial depth, and burial duration were used as fixed effects, while replicates were included as random effects in each model. Seed germination was a probability of 0 or 1; hence, we applied a binomial estimation of the model using a logit link function. The independent-sample *t*-test was used to determine significant differences (or not) in germination percentages between NaCl- and PEG-treated seeds at the same osmotic potential. Duncan’s multiple range tests were used to compare means when significant differences were found. All analyses were conducted using the SPSS 21 software (SPSS Inc., Chicago, IL, United States).

## Results

### Seed Mass

Thousand seed weight of seed lots A and B of *E. sativa* was 1.64 ± 0.02 g and 1.94 ± 0.05 g, respectively.

### Effect of Temperature and Light on Germination

**FIGURE 2:**
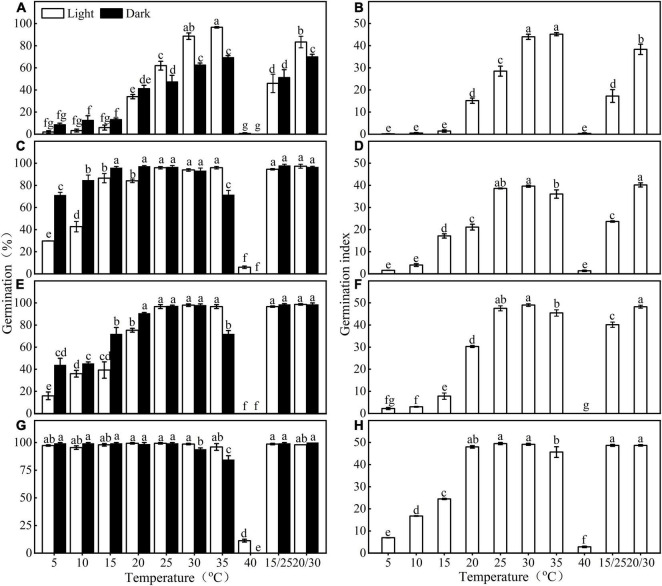
Effect of temperature and light on germination percentage and germination index of fresh **(A,B,E,F)** and stored **(C,D,G,H)** *Eruca sativa* seeds of both seed lots. a, b, c, and d are seed lot **(A)**; e, f, g, and h are seed lot **(B)**. Error bars are ± 1 SE, *n* = 3. Different lowercase letters indicate significant difference (*P* < 0.05) across all temperature regimes and light conditions.

Germination of *E. sativa* seeds varied with incubated temperature, photoperiod condition, and dry storage (Figure 2 and Table 1). Germination percentage and GI first increased and then decreased as temperature increased from 5 to 40°C, in both photoperiod conditions. Fresh seeds of seed lot A only reached high germination (>85%) at 30 and 35°C in light, but after 1 year of storage, seeds achieved high germination under a wider temperature range (15–35°C in light, 10–30°C in continuous darkness; Figures 2A,B). The effect of light on germination varied with the incubated temperature. At a low temperature (<20°C), light inhibited germination, but at high temperatures, light significantly promoted germination.

**TABLE 1:** Effect of light, incubation temperature, storage, and their interaction on germination percentage of *Eruca sativa* using a generalized linear mixed model (GLMM).

Seed lots	Sources of variation	Wald statistic	d.f.	Wald/d.f.	Chi pr
Seed lot A	Storage (ST)	447.11	1	447.11	<0.001
	Light(L)	20.67	1	20.67	<0.001
	Temperature(T)	1676.37	9	186.26	<0.001
	ST*L	34.47	1	34.47	<0.001
	ST*T	410.18	9	45.58	<0.001
	L*T	324.88	9	36.1	<0.001
	ST*L*T	9.67	9	1.07	0.391
Seed lot B	Storage (ST)	167.49	1	167.49	<0.001
	Light(L)	33.83	1	33.83	<0.001
	Temperature(T)	768.53	9	85.39	<0.001
	ST*L	61.62	1	61.62	<0.001
	ST*T	112.31	9	12.48	<0.001
	L*T	75.68	9	8.41	<0.001
	ST*L*T	8.7	9	0.97	0.474

*The asterisk refers to the interaction effect between variables before and after the asterisk (n = 3).*

Seed lot B showed a similar trend with seed lot A, but the widening of the temperature range where seeds reached high germination (>85%) was more obvious than that of seed lot A. After 1 year of storage, except for 40°C, high germination was reached at all temperatures (Figures 2E,G).

### Effect of Water and Salinity Stress on Germination

**FIGURE 3:**
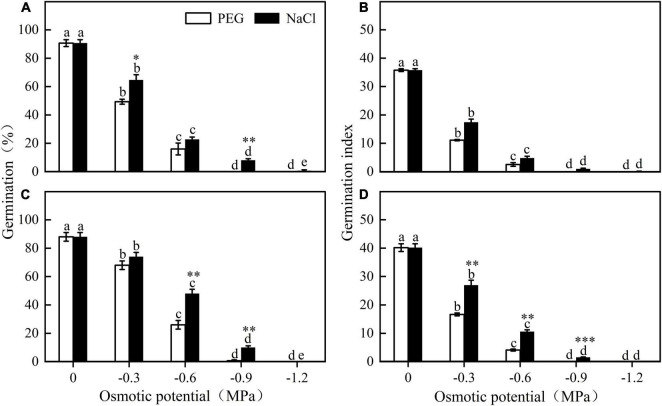
Germination percentage and germination index of *E. sativa* seeds under different potential iso-osmotic NaCl and polyethylene glycol (PEG) solution. **(A,B)** are seed lot **(A)**, and **(C,D)** are seed lot **(B)**. Error bars are ± 1 SE, *n* = 3. Different lowercase letters indicate significant difference (*P* < 0.05) among different osmotic potential under same treatment. Bar with asterisk indicates significant difference between germination percentages of seeds treated with NaCl and PEG solution in the same osmotic potential. *, ^**^, and ^***^ mean significant at 0.05, 0.01, and 0.001 levels, respectively.

Germination of different seed lots shared the same response trend to water stress. Seed germination percentage and index decreased significantly with decreasing osmotic potential. At a given osmotic potential, seed germination percentage and index were higher in NaCl than in PEG. For example, when the osmotic potential was -0.6 MPa, germination of seed lot B in NaCl and in PEG was 48 ± 3.06% and 26 ± 3.06%, respectively (Figure 3).

### Seedling Emergence

**FIGURE 4:**
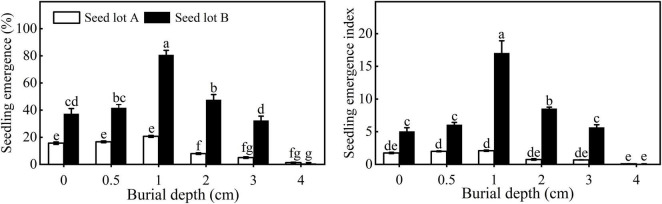
Seedling emergence and emergence index of *E. sativa* sowed at different depths. Error bars are ± 1 SE, *n* = 3. Different lowercase letters indicate significant difference (*P* < 0.05) among different burial depths.

Seedling emergence and EI of both seed lots first increased and then decreased with increasing sowing depth. The highest seedling emergence of seed lots A and B was 20.67 ± 0.67% and 80.67 ± 3.38%, respectively, when sown at 1 cm (Figure 4). It is worth noting that there was no significant difference in emergence for seeds buried at 0, 0.5, or 1 cm for seed lot A, whereas for seed lot B, seedling emergence was much higher at 1 cm. Very few seeds emerged when seeds were buried at a depth of 4 cm. Compared with seed lot B, seed lot A had a significantly lower seedling emergence and EI.

### Effect of Dry Storage on Germination

**FIGURE 5:**
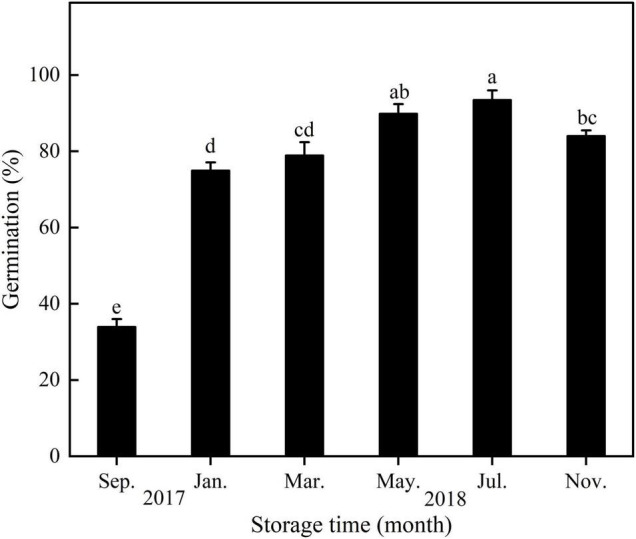
Germination percentage of *E. sativa* seeds during dry storage at 20°C. Error bars are ± 1 SE, *n* = 4. Different lowercase letters indicate significant difference (*P* < 0.05) among different storage times.

With increasing storage time, seed germination of *E. sativa* increased from September 2017 to July 2018 and then decreased (Figure 5).

### Effect of Burial Depth on Persistence of Soil Seed Bank

Burial duration, burial depth, and their interaction significantly affected the percentages of survival and dormancy of *E. sativa* seeds (Table 2). The survival percentage declined with the increased burial duration, and the rate of decline varied with the burial depth. Seeds on the soil surface lost viability very slowly at the beginning of burial, but from June to July, almost all those seeds lost viability. On the contrary, the survival percentage of seeds buried in soil decreased sharply after the first 2 months of burial, and then it decreased gradually with burial duration. After 2 years of burial, 20.67 ± 0.67% and 3.33 ± 0.67% of seeds buried at 5 and 10 cm, respectively, remained viable. Viability of seeds buried at 10 cm decreased more rapidly than that of seeds buried at 5 cm (Figure 1B).

**TABLE 2:** Effect of burial duration, burial depth, and their interaction on percentage of survival and dormancy of *E. sativa* seeds using GLMM.

Seed proportion	Sources of variation	Wald statistic	d.f.	Wald/d.f.	Chi pr
Survival	Depth(D)	154.85	2	77.42	<0.001
	Time(T)	326.06	11	29.64	<0.001
	D*T	48.78	15	3.25	<0.001
Dormancy	Depth(D)	434.35	2	217.18	<0.001
	Time(T)	1525.78	11	138.71	<0.001
	D*T	320.4	15	22.89	<0.001

*The asterisk refers to the interaction effect between variables before and after the asterisk (n = 4).*

For seeds on the soil surface, dormancy was at a low level (<20%) at the beginning of the study, and then it increased to 37.38 ± 4.65% in May 2018. From June to July, all seeds on the soil surface germinated. In contrast, dormancy of seeds in soil exhibited clear cyclical trends. After the burial, dormancy increased in the following spring and early summer. In July 2018, the dormancy of seeds buried at 5 and 10 cm depth reached peaks of 94.66 ± 0.99% and 95.25 ± 0.89%, respectively, and then, dormancy declined until March 2019, reaching the lowest percentages. From March to November 2019, dormancy cycled with time again, but the highest peaks were lower than in the first year (Figure 1C).

## Discussion

### Seed Dormancy and Germination

Fresh seeds of both seed lots reached high germination (>85%) only in a narrow range of high temperature (i.e., 30 and 35°C in light for seed lot A, 30, 35, 15/25, 20/30, and 25°C in light, 20, 30, 15/25, and 20/30°C in continuous darkness for seed lot B). In contrast, after 1 year of dry storage (after ripening), the temperature range for high germination percentages was greatly widened (i.e., 15–35°C in light and 10–30°C in continuous darkness for seed lot A, all temperatures except 40°C for seed lot B) (Figure 2). Further, seeds of *E. sativa* buried at 5 and 10 cm exhibited an annual non-dormancy/dormancy (D/ND) cycle (Figure 1C), thus, fresh seeds of *E. sativa* have non-deep physiological dormancy (NDPD) (Baskin and Baskin, 2014). A significant difference in germination response to temperature and light was observed between two seed lots, suggesting they exhibit different dormancy levels. A possible reason is that seed lot B experienced a longer after-ripening in the field due to the late harvest (Baskin and Baskin, 1985b).

Seed dormancy plays a key role in the timing of germination and seedling emergence of weeds (Hu et al., 2017; Batlla et al., 2020), and thus it affects the probability of seedling survival and the conditions for subsequent plant growth (Donohue et al., 2010). When seeds of *E. sativa* are dispersed in autumn, the existence of conditional dormancy blocked them from germinating at low habitat temperatures; in contrast, dormancy is broken while seeds are cold stratified during winter and thus can germinate in spring. Dormancy break during winter means seeds of *E. sativa* can germinate early at the beginning of the short growing season on the Tibet Plateau, which means the seedlings have the whole growing season for growth and reproduction, especially for annual plants (Stocklin and Baumler, 1996; Mercer et al., 2011; Dubois and Cheptou, 2012; Mondoni et al., 2015). Further, our results showed that after the elimination of dormancy, seeds of *E. sativa* germinated to relatively high percentage at low temperature (Figure 2), suggesting that *E. sativa* is well adapted to the harsh low-temperature environment of the Tibetan Plateau. What is interesting is that the wild populations we used shared similar temperature requirements for germination to the commercial variety, whose base, ceiling, and optimum temperatures for germination are 1, 40.8, and 30°C, respectively (Bakhshandeh et al., 2020). This indicates that *E. sativa* has a wide germination temperature range, which makes it better adapted to a diverse environment.

Soil moisture plays a key role in regulation of seed dormancy release and germination (O’Reilly and De Atrip, 2007; Hu et al., 2017). Our results indicated that germination of *E. sativa* was sensitive to water stress, e.g., most seeds did not germinate at -0.3 MPa. In our study area, more than 80% of the rainfall occurs during the growing season (i.e., April–September), thus, low precipitation in late autumn and early spring may play a key role in blocking seed germination (Figures 1A, 3). Moreover, light inhibited germination of *E. sativa* at low temperatures (<20°C), delaying seed germination on the soil surface until warmer seasonal conditions prevailed (Figures 1C, 2). Thus, the combined effects of exposure to water stress and light may act as a germination avoidance mechanism at the soil surface, which is consistent with the findings for seeds of *Stipa bungeana* (Hu et al., 2013). Further, this mechanism causes different germination times of seeds at different depths, which may be a strategy to reduce competitive pressure and reduce risks (Cao et al., 2012; Wang et al., 2017). However, this strategy is different from that of *Primula alpicola*, which has “skoto-inhibited” seeds (Baskin and Baskin, 2014), resulting in a delay of germination in darkness (Wang et al., 2017).

### Persistence of Soil Seed Bank

Our results indicated that seeds of *E. sativa* on the soil surface formed a transient soil seed bank, but those buried in soil can form at least a short-term persistent soil seed bank, as 20.67 ± 0.67% and 3.33 ± 0.67% of seeds buried in 5 and 10 cm depth remained viable after 24 months burial, respectively (Figure 1B).

The variation in persistence of seeds on the soil surface and those below the surface may be due to differences in the microenvironment at different burial depths (Mennan, 2003; Mennan and Zandstra, 2006; Hu et al., 2017). Burial depths favored Italian ryegrass (*Lolium multiflorum* L.) persistence in the soil seed bank (Cechin et al., 2021) but not that of *Stipa bungeana* (Hu et al., 2014). For *E. sativa* seeds exposed to the soil surface, dormancy was released in winter and remained at a low level in early spring. However, due to light, low temperature and water stress, and their interactions, almost most of the seeds did not germinate until June (Figure 1B), which is consistent with the observation of many seedlings in the field in June. In contrast, when seeds were buried in soil, most of them failed to germinate and were induced into secondary dormancy in spring (Baskin and Baskin, 1980; Fenner and Thompson, 2005; Falavigna et al., 2019). The lack of oxygen (Mennan, 2003), excessive soil moisture (Hu et al., 2017), and small daily temperature fluctuations (Gulden et al., 2004) in deep soil could cause seeds to enter secondary dormancy. Consistent with this, our study clearly showed that seeds of *E. sativa* buried at 5 and 10 cm exhibited an annual D/ND cycle, and this dormancy cycle favored the persistence of soil seed bank. However, seeds dry-stored indoors cannot re-enter secondary dormancy (Figure 1C); this may be due to the more stable temperature and air humidity when seeds were subjected to dry-storage condition (Baskin and Baskin, 2020). The existence of the dormancy cycle and the short-term persistent soil seed bank would make the prevention and control of *E. sativa* more difficult (Batlla et al., 2020).

**FIGURE 6:**
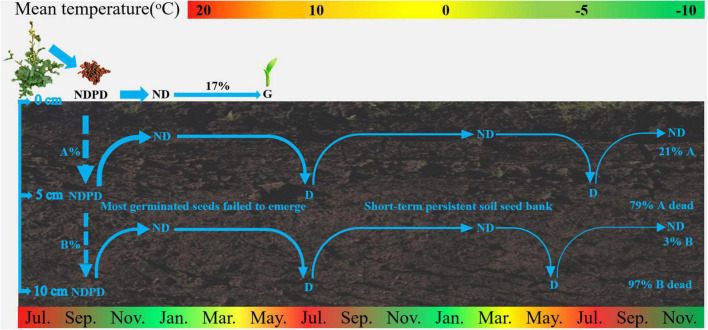
Conceptual models of seed dynamics in populations of *E. sativa*. The percentages indicate proportions of seeds in a soil seed bank that gave rise to seedlings and of those that entered the persistent soil seed bank. NDPD, non-deep physiological dormancy; ND, non-dormancy; G, germination. The broken arrows represent events that may occur, and the width of arrows roughly represents proportion of seeds.

The results of our study on seed bank dynamics of *E. sativa* were summarized in a conceptual model (Figure 6). When dispersed at maturity, seeds have NDPD, and they become ND when exposed to low temperatures in winter. Almost all seeds on the soil surface germinated from June to July. Seeds buried in soil that failed to germinate re-entered to D in spring and early summer, and they exhibited an annual D/ND cycle, which is conducive to the formation of a short-term persistent soil seed bank.

### Implications for Management

For *E. sativa*, seedling that emerged from seeds in the shallow soil from June to July contributed the most to the population of the species, while few seedlings emerged from seeds buried deeper than 5 cm. Thus, we adopted no-tillage to maintain seeds on the soil surface and weeding after the seedling emergence or before the seeds mature to reduce the input of soil seed bank. Furthermore, reducing soil disturbances caused by tillage to keep seeds in deep soil, and most of them will lose their viability in 2–3 years.

## Conclusion

Seeds of *E. sativa* exhibited non-deep physiological dormancy at maturity, which can be broken by dry storage or cold stratification during winter. Burial depth affected the persistence of soil seed bank of *E. sativa*. Seeds on the soil surface formed a transient soil seed bank, but when buried at 5 or 10 cm, seeds exhibited an annual D/ND cycle and can form at least a short-term persistent soil seed bank. The interaction of dormancy and germination with the environment formed an effective survival strategy that blocked seeds from germinating in autumn but promoted it in early spring, thereby increasing the chances of seedling survival. Seedling that emerged from seeds in the shallow soil from June to July contributed the most to the population. No-tillage would be a good management strategy for *E. sativa*.

## Data Availability Statement

The raw data supporting the conclusions of this article will be made available by the authors, without undue reservation.

## Author Contributions

X-WH conceived this topic and revised the first draft of the manuscript. J-JW, C-ZJ, and D-LC performed the material preparation, data collection, and analysis. C-ZJ wrote the first draft of the manuscript. All authors commented on previous versions of the manuscript, read, and approved the final manuscript.

## Conflict of Interest

The authors declare that the research was conducted in the absence of any commercial or financial relationships that could be construed as a potential conflict of interest.

## Publisher’s Note

All claims expressed in this article are solely those of the authors and do not necessarily represent those of their affiliated organizations, or those of the publisher, the editors and the reviewers. Any product that may be evaluated in this article, or claim that may be made by its manufacturer, is not guaranteed or endorsed by the publisher.
